# Proteasome and Ribosome Ubiquitination in Retinal Pigment Epithelial (RPE) Cells in Response to Oxidized Low-Density Lipoprotein (OxLDL)

**DOI:** 10.3390/biomedicines13123004

**Published:** 2025-12-08

**Authors:** Francesco Giorgianni, Sarka Beranova-Giorgianni

**Affiliations:** Department of Pharmaceutical Sciences, The University of Tennessee Health Science Center, Memphis, TN 38163, USA

**Keywords:** retinal pigment epithelium, oxidized LDL, drusen, proteome, oxidative stress, proteostasis, ubiquitin, UPS, proteasome, ribosome

## Abstract

**Background/Objectives:** Oxidative stress plays a significant role in the development and progression of age-related macular degeneration (AMD). Retinal pigment epithelium (RPE) cells are specialized multifunctional cells indispensable for the maintenance of vision. The dysfunction and death of RPE cells in the macula characterize the onset and development of AMD. Of the various toxic agents that impact the health of the RPE, particular focus has been given to various forms of lipoproteins and their cytotoxic derivatives normally present in the retina. Oxidized low-density lipoprotein (OxLDL), derived from LDL in a pro-oxidative environment, is found adjacent to RPE cells as part of drusen, extracellular deposits that are a hallmark feature of AMD. OxLDL is a potent inflammatory agent and it has been implicated in cardiovascular and neurodegenerative conditions. The cellular molecular mechanisms triggered by OxLDL are only partially understood. The focus of this study was to characterize changes in the proteome of RPE cells after exposure to OxLDL, with a focus on the characterization and quantification of ubiquitinated proteins. **Methods:** Identification and quantification were performed with a high-resolution LC-MS/MS-based proteomics workflow after immune-enrichment for ubiquitinated peptides. **Results:** In total, out of the more than 1000 RPE ubiquitinated peptides quantified, OxLDL treatment caused a significant increase in ubiquitinated peptides compared to LDL and untreated cells. Principal component analysis (PCA) of the differentially ubiquitinated proteins (265) reduced the data complexity in two main groups of variables (proteins). **Conclusions:** Gene ontology enrichment analysis of the grouped proteins with the highest loading contribution to principal component 1 (PC1) and principal component 2 (PC2) revealed significant ubiquitination changes upon OxLDL treatment in proteins of the ubiquitin–proteasome system (UPS) responsible for proteasome-mediated catabolic processes and in protein members of the cellular translation machinery.

## 1. Introduction

Proper synthesis, folding, trafficking, and clearance of proteins are essential for cell function [1]. Maintenance of a functional and balanced proteome within the cell is referred to as protein homeostasis or proteostasis, and it is enabled by a complex and integrated quality control network comprising several thousand components responsible for the synthesis of properly folded proteins, maintenance of their conformational stability, and for the degradation of damaged or misfolded proteins [2]. Broadly speaking, the proteostasis network is organized into three functional branches responsible for (a) control of protein synthesis, (b) adoption and maintenance of proper protein folding, and (c) protein degradation. The components of protein synthesis include ribosomes, factors involved in each stage of translation (initiation, elongation, and termination), and the ribosome-associated quality control (RQC) system. Molecular chaperones and co-chaperones are components central to proper protein folding and maintenance of conformational stability [3]. The chaperone systems aid in the folding of newly synthesized polypeptides, assist in the assembly of multiprotein complexes, facilitate refolding of misfolded proteins, and prevent protein aggregation. The protein degradation branch of the proteostasis network comprises the ubiquitin–proteasome system (UPS) [4] and the autophagy–lysosomal pathway (ALP) [5]. Regulated protein degradation is an important part of protein turnover to maintain and adjust appropriate levels of functional proteins, and it is also a key mechanism for the removal of damaged proteins to avoid accumulation of potentially cytotoxic proteins and protein aggregates [6]. The UPS is responsible for the majority of protein turnover, and it often cooperates with molecular chaperones [7]. To target proteins for degradation, the proteins are covalently modified by the attachment of ubiquitin, and ubiquitination serves as the recognition signal for protein degradation by the 26S proteasome complex of the UPS [8].

The retinal pigment epithelium (RPE) are highly specialized, multifunctional cells located adjacent to the photoreceptor cells of the macula [9]. The RPE forms a monolayer of polarized cells, where the apical side faces the photoreceptors, while the basal surface is in contact with the Bruch’s membrane of the choroid. The RPE cells play a crucial role in supporting the function and health of the photoreceptors, and they are vital for normal vision [10]. Damage or dysfunction of the RPE may lead to age-related macular degeneration (AMD), a progressive retinal disease that usually affects older adults, leading to loss of central vision. AMD is the leading cause of vision loss in the elderly in developed countries [11]. Oxidative stress and related damage to molecular components of the RPE have been linked with the onset and progression of AMD, especially with dry AMD, which is the prevalent form of the disease [12]. Accumulation of oxidized lipids and proteins contributes to the formation of drusen, extracellular deposits present between the RPE and Bruch’s membrane that are characteristic clinical features of AMD [13]. Studies have shown that protein misfolding and aggregation are key clinical hallmarks in most age-related, neurodegenerative diseases [14]. Studies in cultured RPE cells have shown that conditions of oxidative stress like exposure to A2E-mediated photooxidation or to H_2_O_2_ increase ubiquitination activities and inactivation of the proteasome [15].

We have recently reported results of a global proteomics study of the effects of OxLDL on RPE cells [16]. Our investigation unveiled that exposure of RPE cells to non-cytotoxic levels of OxLDL triggered a complex response characterized by significant alterations in the expression of *ca.* 300 proteins that belonged to multiple functional modules. OxLDL treatment resulted in changes in proteins involved in antioxidant defense, glucose metabolism, and lipid metabolism. The most notable and previously unreported findings were that OxLDL led to a marked downregulation of ribosomal and translation initiation proteins, indicating inhibition of protein synthesis. Simultaneously, there was an upregulation of proteins involved in autophagy, suggesting enhanced degradation of misfolded or damaged proteins. This coordinated shift implies that RPE cells mitigate OxLDL-induced oxidative stress by reducing new protein production and promoting intracellular clearance mechanisms. These findings indicate involvement of the proteostasis network components as major molecular mechanisms through which the RPE cells respond to OxLDL-induced oxidative stress.

Significant to the results presented here, it has been shown that under a heavy load of misfolded proteins, proteasome activities decrease and the proteins comprising the proteasome undergo ubiquitination, followed by degradation through the autophagy–lysosomal pathway [17].

To expand on our novel findings of proteome adaptations employed by the RPE to mitigate disruptions in proteostasis following exposure to OxLDL, here we report results from a pilot analysis of the effects of OxLDL on the subset of ubiquitinated proteins in the RPE proteome. Using tailored enrichment of ubiquitinated species in combination with quantitative high-resolution LC-MS/MS, we show that OxLDL treatment results in increased protein ubiquitination, manifested as an increase in the total number of ubiquitinated and poly-ubiquitinated peptides in the OxLDL-exposed RPE cells. Bioinformatics analysis of the differentially ubiquitinated proteins highlighted changes in the ubiquitination status of proteins of the UPS, which functions in the degradation/clearance of misfolded and damaged proteins. Furthermore, changes were uncovered in the ubiquitination of ribosomal proteins, which are associated with protein translation.

## 2. Materials and Methods

### 2.1. Cell Culture and Treatment

ARPE-19 cells were cultured according to well-established protocols in our laboratory and based on methods developed by Hazim et al. [18]. Specifically, cells were maintained in nicotinamide-containing media and grown on Transwell cell culture inserts to allow for the establishment of polarized RPE monolayers with fully developed junctional structures and RPE-specific protein markers, e.g., RPE65 [19,20]. The improved growth methods, which use nicotinamide as an essential differentiation promoting factor, allow to obtain in a relatively short period of time (*ca.* 2 weeks) cells which exhibit proper cell morphology and cytoskeletal organization [18]. Prior to OxLDL treatment, the ARPE-19 cells were maintained in serum-free medium for 24 h and then treated with LDL (100 µg/mL) or OxLDL (100 µg/mL) for 24 h. Previously, we have determined that there is minimal cytotoxicity (<5% cell death) after 24 h exposure of ARPE-19 cells to OxLDL under these conditions [16,21]. All experiments were performed in multiple biological replicates (*n* = 3) per treatment group. To validate cellular response to OxLDL treatment, the increased expression of one of the top upregulated proteins, heme oxygenase-1 (HO-1 or HMOX1), was performed by Western blot analysis, as previously described [21].

### 2.2. Sample Preparation and Protein Digestion

Protein digestion and peptide purification were performed with a commercial protein digestion and purification kit from Protifi (Fairport, NY, USA) following the manufacturer’s recommendations with minor modifications as described. The Protifi protocol is based on the suspension TRAPping filter (sTRAP) method [22]. Briefly, differentiated ARPE-19 cells were collected from culture wells, washed with PBS buffer (pH 7.4) to remove excess growth medium, and centrifuged at 3000 × *g* for 5 min. The cell pellets were suspended in 250 µL of 2× lysis buffer (100 mM triethylammonium bicarbonate (TEAB) pH 7.6, containing 10% SDS). Protein content was measured with the micro BCA kit with BSA as a protein quantification standard (Thermo Fisher Scientific, Waltham, MA, USA). Sample total protein content ranged from 1.3 to 1.5 mg. Samples were reduced with 10 µL of DTT solution (1 M in 50 mM TEAB buffer, pH 7.6, 5% SDS) at 55 °C for 30 min. Alkylation of cysteine residues was performed by the addition of 20 µL of iodoacetamide (2.0 M in 50% EtOH-H_2_O) at room temperature in the dark for 15 min. Samples were acidified by the addition of 60 µL of 12% phosphoric acid and diluted with 3.3 mL of S-Trap buffer (90% MeOH, 100 mM TEAB, pH 7.1). The diluted sample mixes were added to the kit-provided spin columns. The columns were spun for 30 s at 3200 × *g*. The flow-through was removed and the columns were washed 3 times with 3 mL of S-Trap buffer. Digestion buffer (50 mM TEAB, pH 8.0 containing 30 µg of sequencing-grade trypsin (Promega, Madison, WI, USA) per mL) was added to each sample (350 µL). Samples were incubated at 37 °C in an incubator oven overnight. Peptides were eluted sequentially with 500 µL of 50 mM TEAB (added into the spin columns before the first centrifugation), 500 µL of dH_2_O, 0.2% formic acid (FA), and 500 µL of 50% ACN-H_2_O, 0.2% FA. The combined fractions were dried in a speed vacuum centrifuge after pH adjustment to *ca.* 3.0 with the addition of 10 µL of FA.

### 2.3. Enrichment of Ubiquitinated Peptides

ARPE-19 cell samples were processed with the PTMScan ubiquitin enrichment kit from Cell Signaling (cat. # 34608; Danvers, MA, USA), which is based on sample enrichment of K-GG peptides by immunoaffinity purification of protein digests [23]. The dry peptides were dissolved in 1.5 mL of binding buffer (provided by the kit manufacturer), and the pH was adjusted from 6.5 to 7.0 with the addition of 20 µL per sample of 1 M Tris buffer. The remaining steps involved in the enrichment process followed exactly the manufacturer’s recommendations. After elution (*ca.* 100 µL), the sample’s volume was reduced in a speed vacuum centrifuge to *ca.* 40 µL. Finally, the peptides were desalted by micro C18-containing cartridges (Ziptip, MilliporeSigma, Burlington, MA, USA), dried, and stored at −80 °C until LC-MS/MS analysis was performed.

### 2.4. LC-MS/MS Analysis

Sample tryptic peptides (5 µL dissolved into 97% H_2_O—3% ACN—0.1% TFA) were loaded onto a nano flow trap column (Acclaim PepMap 100, 75 µm × 20 mm—C18, 3 µm, 100 Å—Thermo Fisher Scientific) and separated on an Acclaim PepMap RSLC nano flow column (75 µm × 500 mm—C18, 2 µm, 100 Å—Thermo Fisher Scientific) with a linear gradient of H_2_O-ACN containing 0.1% (*v*/*v*) formic acid and a flow rate of 300 nL/min. Eluted peptides were identified and quantified on an orbitrap MS instrument (Fusion Lumos—Thermo Fisher Scientific) set in data-dependent acquisition mode with MS resolving power of 120,000 (FWHM, at 200 *m*/*z*). The MS2 settings were 0.7 *m*/*z* quadrupole isolation window, HCD activation of 30%, and orbitrap resolving power of 30,000 (FWHM at 200 *m*/*z*) with 30 s dynamic exclusion.

### 2.5. Mass Spectrometry Data Processing

Peptides and proteins were identified with the search engine Sequest HT (Thermo Fisher Scientific) using a human database (SwissProt, TaxID 9606 (*Homo sapiens*), v.2017-10-25) and reverse decoy target database (concatenated). The searches allowed for 2 trypsin missed cleavages. The total dynamic modifications per peptide were set as follows: 3 maximum equal and 4 maximum dynamic modifications per peptide. Peptide dynamic modifications included methionine oxidation (+15.995 Da) and lysine ubiquitination (GG tryptic remnant, +114.043 Da). Peptide static modifications included cysteine carbamidomethylation (+57.021 Da). Dynamic protein modifications (protein terminus) included N-terminal acetyl modification (+42.011 Da); N-terminal methionine loss (−131.040 Da); and N-terminal methionine loss plus acetyl (−89.030 Da). Precursor and fragment ion mass tolerance was set to 10 ppm and 0.02 Da, respectively. Validation and filtering at the PSM (peptide spectrum matching) level (*q* value) were performed by Percolator with an FDR ≤ 0.01. The identification of proteins or protein groups required at least one validated peptide sequence unique to a protein or a protein group (strict parsimony principle applied). Criteria for label-free quantification of proteins and peptides (precursor ion area detection) included peptide uniqueness (plus Razor) and MS peak area (minimum S/N threshold: 5). Peptides were normalized by total peptide amounts. Peptide abundance was calculated by the abundance sum of the assigned PSMs. Protein abundance was calculated by the summed abundances of the assigned peptides.

### 2.6. Statistical Analysis

Protein abundance values were analyzed with R, using R Limma linear regression and Ebayes function, to calculate the F values and adjusted pairwise *p*-values. The fold changes were calculated with the ratio of the averages in the test conditions divided by the control values. If the ratio value was less than 1, i.e., the control was larger, the negative inverse of the fold change was calculated. Principal component analysis (PCA) was performed with GraphPad (v. 10, Boston, MA, USA). To select the most significant loadings that contribute to the corresponding score, a cut-off of |0.5| was used.

## 3. Results

**Figure 1:**
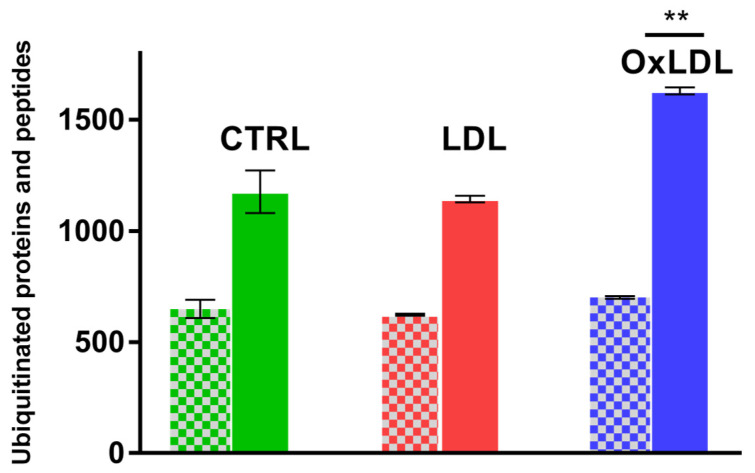
OxLDL treatment increases the number of ubiquitinated peptides (solid bars), not proteins (checkered bars). Global ubiquitome analysis of differentiated ARPE-19 cells following 24 h exposure to 100 µg/mL of LDL or OxLDL (** *p* < 0.01; *n* = 3).

**Figure 2:**
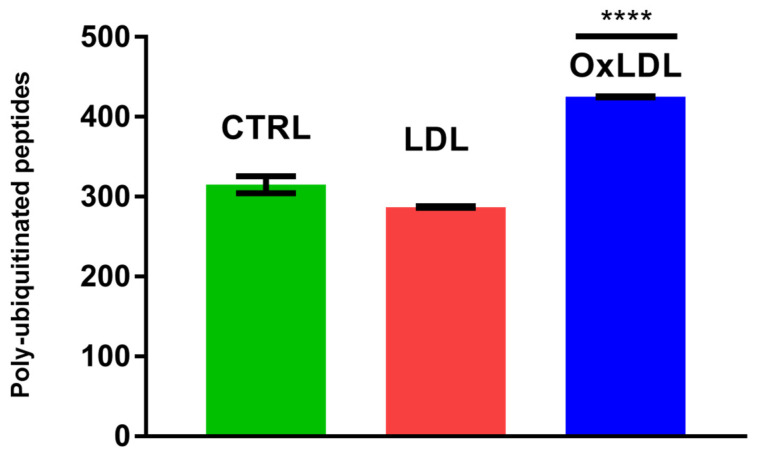
OxLDL treatment increases the number of poly-ubiquitinated peptides. Global ubiquitome analysis of ARPE-19 cells following 24 h exposure to 100 µg/mL of LDL or OxLDL. **** *p* < 0.0001 (*n* = 3).

To characterize changes in protein ubiquitination after OxLDL and LDL treatments, we analyzed the ARPE-19 cell ubiquitome by high-resolution mass spectrometry. Differentiated ARPE-19 cells were treated for 24 h with either vehicle control (PBS solution) or 100 µg/mL of LDL or OxLDL. Whole-protein extracts were digested with trypsin and enriched for ubiquitinated peptides by immunoaffinity methods. The ubiquitinated peptide-enriched samples were analyzed with an Orbitrap Fusion Lumos. The LC-MS/MS analysis identified more than 600 ubiquitinated proteins and more than 1000 ubiquitinated peptides per dataset (Supplementary Table S1). Biostatistical analysis of the data showed a significant increase in the ubiquitination status of 265 proteins, mostly in the OxLDL-treated group. Interestingly, the overall number of ubiquitinated proteins did not change significantly between the controls and LDL- or OxLDL-treated samples (Figure 1). However, a group of 265 proteins contained a significant increase in ubiquitinated peptides, both in terms of absolute quantity and in the number of ubiquitination sites. The biological group with the largest increase in ubiquitinated peptides was represented by the OxLDL-treated samples (Figure 1). Significantly, we observed a dramatic increase in poly-ubiquitinated peptides in OxLDL-treated samples (Figure 2).

**Figure 3:**
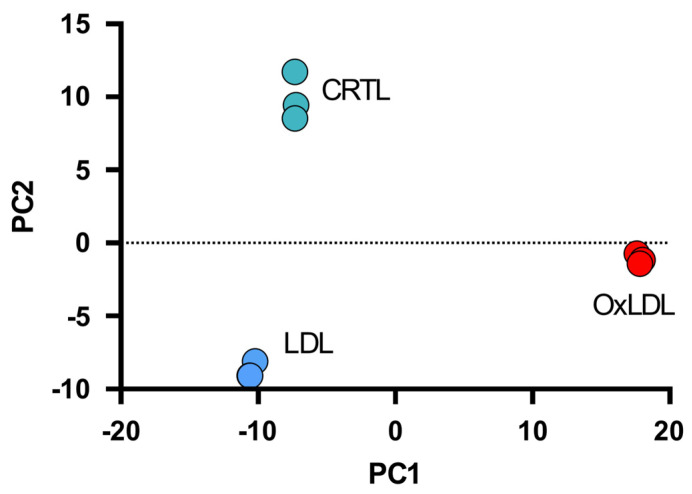
Principal component analysis (PCA) of differentially ubiquitinated proteins. Scores of the first principal component (PC1) and second (PC2) of ubiquitinated proteins (265), significantly different in ubiquitinated peptides abundances (*p* < 0.05), between CRTLs and treated samples. OxLDL-treated samples contribute the most to the PC1 score value.

Principal component analysis of the 265 most significantly differentially ubiquitinated proteins (F *p*-values < 0.01 and FC > |1.5|) could reduce the complexity of the dataset into two main components (PC1 and PC2), which, when combined, could account for 94% of the sample’s variability (Figure 3). The most significant principal component (PC1) could account for 69% of the observed variation, while PC2 could account for 25%. As shown in Figure 3, the OxLDL-treated samples contributed the most to PC1. Analysis of the most significant loadings (>|0.5|) for PC1 (70 proteins) with DAVID functional annotation clustering tool [24] generated the highest statistically significant gene ontology biological process (GOTERM_BP_DIRECT) score for the “*proteasome-mediated ubiquitin-dependent protein catabolic process*” cluster and the highest statistically significant gene ontology cellular component score (GOTERM_CC_DIRECT) for the “*proteasome complex*” cluster (Table 1). A second ubiquitinated protein list which included proteins with the largest loadings (>|0.5|) for PC2 analyzed using the same biostatistical tool yielded the highest statistically significant GOTERM_BP_DIRECT score for the “*cytoplasmic translation*” cluster and the highest GOTERM_CC_DIRECT score for the “*cytosolic ribosome*” cluster (Table 2). The ubiquitome analysis of ARPE-19 cells after OxLDL-induced oxidative stress points to the activation of two major biological processes: proteasome-mediated catabolic activity and protein translation.

## 4. Discussion

Eukaryotic cells have evolved stress response mechanisms to cope with proteostatic disruptions. The accumulation of misfolded and unfolded proteins within the endoplasmic reticulum (ER) triggers the unfolded protein response (UPR) [25], which can restore normal protein homeostasis by removing proteins that lack the correct three-dimensional structure through activation of the proteolytic machinery of the ubiquitin–proteasome system (UPS) [26]. Activation of the UPR and degradation of misfolded proteins by the UPS is one element of a complex cellular mechanism that aims to reestablish normal physiological conditions after an acute stress event. A second cellular protective mechanism, broadly functioning across other cellular compartments, is the integrated stress response (ISR) [27], which allows cells to adapt to various types of stress by modulating overall protein synthesis via eIF2α phosphorylation [28], a key initiation factor for translation, and by the increased production of molecular chaperones [29]. Under acute stress conditions, the ISR can halt protein synthesis [30]. These protective responses, activation of the UPS and ISR, involve multifaceted signaling pathways that ultimately lead to increased protein degradation and decreased ribosome activity and protein synthesis. If these mechanisms are overwhelmed, cells try to reestablish proteostasis by activating the autophagy–lysosomal pathway (ALP), which clears all affected organelle multiprotein complexes, including the proteasome [31]. When activation of ALP fails to restore normal physiological conditions, cells enter apoptosis [32]. In this study, several protein components of the 26S proteasome complex were found to have increased ubiquitination (Table 1). Almost all the proteins listed in Table 1 are an integral part of the 19S regulatory component of the proteasome, also known as the regulatory particle (RP). The proteasomal ubiquitin receptor (ADRM1/Rpn13) is part of the RP of the 26S proteasome [33]. It binds ubiquitin-tagged proteins targeted for degradation and facilitates their transfer into the proteolytic complex of the proteasome (20S) [34]. A second essential component of the RP subunit of the proteasome is the non-ATPase regulatory subunit 4 (PSMD4/Rpn10) [35]. It has been shown that inhibition of PSMD4/Rpn10 causes accumulation of ubiquitinated proteins, cell cycle arrest, and apoptosis [36].

A group of six ATPases (Rpt1 through Rpt6) form an hexameric ring at the base of the RP subunit of the proteasome. The interactions of the C-termini of these ATPases with the heptameric α ring of the core particle allow ubiquitinated proteins targeted for degradation to move inside the proteolytic core of the proteasome [37]. Three of the six ATPases were characterized as differentially ubiquitinated in our study. Interestingly, increased levels of one of these ATPases, PSMC1/Rpt2 regulatory subunit 4 (Table 1), were found in the aqueous humor of AMD patients [38].

The 26S proteasome non-ATPase regulatory subunit 10 PSMD10/gankyrin (O75832) is an oncoprotein overexpressed in most hepatocellular carcinomas. Gankyrin is known to interact with one of the RP ATPase subunits (Rpt4). The association of gankyrin with Rpt4 is critical for the degradation pathway of the retinoblastoma protein (pRb), a tumor suppressor protein [39].

The remaining two proteins in Table 1, the heat shock protein beta-1 (HspB1) (P04792) and thioredoxin-like protein 1 (TXNL1) (O43396), while not an integral part of the proteasome, are both functionally associated with components of the RP of the proteasome. HspB1 is a molecular chaperone that, once activated by phosphorylation, plays an important protective role against oxidative stress [40,41]. Thioredoxin-like protein 1 plays the dual role of a disulfide oxidoreductase and a redox-active cofactor of the RP. It binds to Rpn11, a subunit of the RP, and facilitates transport of ubiquitinated proteins to the proteasome proteolytic core [42,43].

Our data show a significant increase in ubiquitination in all these proteins critical for the regulatory function of the proteasome upon OxLDL treatment. For some of the proteins listed in Table 1, there is evidence that an increase in ubiquitination under intense proteotoxic stress is associated with the disassembly of the entire 26S proteasome particle and its proteolytic degradation by autophagocytic mechanisms that often precede cell death by apoptosis [44]. Little is known about the RPE-specific ubiquitination processes that lead to cell death after the 26S proteasome is overwhelmed by acute oxidative stress events. However, based on the work of other authors, we believe that our findings are relevant to the field of age-related macular degeneration (AMD) [45].

Our ubiquitome data analysis of ARPE-19 cells after OxLDL-induced oxidative stress points to the activation of two major biological processes: proteasome-mediated catabolic activity and ribosome-dependent protein translation. Other studies have shown the presence of ubiquitin and other proteasome subunits in the aqueous humor of AMD patients [46,47].

The second set of differentially ubiquitinated proteins of potential mechanistic significance uncovered in our study are RPE proteins associated with ribosomal function. Ribosomes are large ribonucleoprotein complexes responsible for protein synthesis, and they are closely involved in the cells’ response to oxidative stress. Ribosomal function and dynamics may be significantly affected by stress-induced proteostatic disruptions. In our previous differential expression proteomics work, OxLDL treatment of the RPE cells resulted in a decreased abundance of a large number of ribosomal proteins from both the 60S and 40 ribosomal subunits [16]. In our ubiquitination study, six ribosomal proteins were found to be differentially ubiquitinated upon OxLDL exposure (Table 2).

Evidence has emerged to show that in addition to serving as a signal for proteasomal degradation, ubiquitination/deubiquitination also functions as a regulatory modification, similarly to phosphorylation/dephosphorylation. In this manner, ubiquitination plays a role as a signaling moiety in non-degradative processes that involve the ribosomes [48,49]. Ribosome stalling refers to the interruption of translation that occurs when a ribosome is impeded on an mRNA transcript, often due to structural abnormalities in the mRNA or defects in the nascent polypeptide [50]. Under oxidative stress, reactive oxygen species can damage mRNA, ribosomal proteins, or nascent peptide chains, which increases the likelihood of stalling. This activates quality control mechanisms, including the ribosome-associated quality control (RQC) pathways, which detect stalled ribosomes and facilitate their resolution or degradation. Ribosome ubiquitination plays a role in the RQC [48,49,51,52]. It has been reported that RQC-mediated resolution of stalled ribosomes is modulated by the ZNF598 ubiquitin ligase and the 40S ribosome-associated protein RACK1 [52]. These factors participated in the ubiquitination of specific 40S ribosomal proteins: ZNF598 ubiquitinated the RPS10 and RPS20, while RACK1 facilitated ubiquitination of RPS2, RPS3, and RPS20. It was suggested that ZNF598- and RACK-1-mediated regulatory ubiquitination of these subsets of 40S ribosomal proteins are early events required for recognition by factors recruited to the stalled ribosomes for subsequent RQC reactions.

In the work presented here, OxLDL induced changes in the ubiquitination of four components of the 60S ribosomal subunit: uL1 (RPL10A), uL4 (RPL4), eL8 (RPL7A), and uL6 (RPL9). Furthermore, two differentially ubiquitinated proteins from the 40S ribosomal subunit were found: eS8 (RPS8) and RACK1. Regulatory ubiquitination of components of the 40S subunit has been shown to be of importance in the RQC pathway [52], including the regulation of protein translation machinery in response to stress [53,54]. RACK1 is a scaffolding protein associated with the 40S ribosome, and it has been shown to facilitate regulatory ubiquitination of the 40S subunit [52]. Ubiquitination of RACK1 itself has been reported in yeast under stress conditions [53].

Taken together, our results indicate that ubiquitin-mediated mechanisms play a role in the response of RPE cells to perturbations of proteostasis induced by OxLDL. It is likely that the dynamics of RPE ribosome ubiquitination reflect a complex scenario in which attachments of the ubiquitin moiety may direct the protein for proteasome-mediated degradation, and/or a subset of the ubiquitination may be regulatory in nature.

## 5. Conclusions

In summary, disruption of proteostasis can affect various cellular organelles and be a major contributor to the onset and development of numerous neurodegenerative disorders [55]. In this study, we analyzed the molecular responses of an RPE-derived cell line to a sudden intracellular increase of a powerful oxidizing agent such as OxLDL. Specifically, we focused on the effects of OxLDL on the ubiquitome both qualitatively and quantitatively. Our data show that upon treatment, the ubiquitome is characterized by significant changes that involve hundreds of proteins. Gene ontology and statistical analysis of the data indicate that major changes occur within two critical multiprotein complex structures: the proteasome and the ribosome. Protein components of the 26S proteasome, in particular members of the 19S regulatory subunit, are impacted the most. Based on current knowledge, our data suggest that upon OxLDL treatment, there is an increase in ubiquitination within the proteasome which results in the proteasome becoming a target for degradation by the ALP system. Oxidized low-density lipoproteins are commonly present in the retina. It is therefore reasonable to suggest that one of the negative impacts of these oxidizing agents in vivo is that they cause dysfunction of the UPS degradation machinery and possibly its degradation.

At the same time, the oxidized low-density lipoproteins present in the retina can impact the ribosomes in multiple ways, including through direct interference with protein synthesis, activation of stress responses that alter ribosomal activity, changes in the translation machinery composition, and impairment of ribosome quality control pathways.

These effects, on the proteasome and the ribosome, contribute to cellular dysfunction and are implicated in various diseases associated with proteostatic imbalance, including neurodegenerative disorders [56,57,58] and age-related conditions like AMD where RPE function is compromised [59].

In this study, we report on qualitative and quantitative changes in the ubiquitination status of numerous proteasome and ribosome protein components. Understanding how exactly proteasome and ribosomes functions are modulated during oxidative stress and whether this process is passive or actively regulated by specific signals remains an area ripe for exploration.

## Figures and Tables

**Table 1:** Differentially ubiquitinated proteins in OxLDL-treated ARPE-19 cells associated with proteasome function.

Accession Number	Protein Name	Adjusted *p*-Value	FC OxLDL Versus CRTL	# Ubiquitinated Peptides	# Ubiquitinated Residues	PC1	PC2
Q16186	Proteasomal ubiquitin receptor (ADRM1/Rpn13)	2.32 × 10^−5^	1.53	2	2	0.979	0.096
P04792	Heat shock protein beta-1 (HspB1)	1.07 × 10^−8^	4.23	4	5	0.795	−0.609
P55036	26S proteasome non-ATPase regulatory subunit 4 (PSMD4/Rpn10)	3.93 × 10^−6^	1.58	4	6	0.948	0.292
P62191	26S proteasome regulatory subunit 4 (PSMC1/Rpt2)	3.79 × 10^−6^	2.83	7	7	0.989	−0.011
P35998	26S proteasome regulatory subunit 7 (PSMC2/Rpt1)	4.01 × 10^−7^	4.25	8	9	0.992	−0.051
P62333	26S proteasome regulatory subunit 10B (PSMC6/Rpt4)	7.49 × 10^−9^	10.31	9	12	0.982	−0.173
O75832	26S proteasome non-ATPase regulatory subunit 10 (PSMD10/Gankyrin)	6.36 × 10^−8^	2.82	1	1	0.997	0.008
O43396	Thioredoxin-like protein 1 (TXNL1)	5.82 × 10^−12^	inf	4	4	0.991	−0.129

The # symbol represents “number of”.

**Table 2:** Differentially ubiquitinated proteins in OxLDL-treated ARPE-19 cells associated with ribosomal function.

Accession Number	Protein Name	Adjusted *p*-Value	FC OxLDL Versus CRTL	# Ubiquitinated Peptides	# Ubiquitinated Residues	PC1	PC2
P63244	Small ribosomal subunit protein (RACK1)	2.04 × 10^−2^	1.50	2	2	0.246	−0.718
P62906	Large ribosomal subunit protein (uL1)	3.75 × 10^−8^	3.33	2	2	0.817	−0.568
P36578	Large ribosomal subunit protein (uL4)	6.77 × 10^−3^	−4.32	2	2	−0.815	−0.508
P62424	Large ribosomal subunit protein (eL8)	7.53 × 10^−4^	−1.63	3	3	−0.747	−0.657
P32969	Large ribosomal subunit protein (uL6)	4.12 × 10^−3^	1.88	1	1	−0.341	−0.922
P62241	Small ribosomal subunit protein (eS8)	5.41 × 10^−4^	−1.92	1	1	−0.701	−0.708

The # symbol represents “number of”.

## Data Availability

The original contributions presented in this study are included in the article/Supplementary Material. Further inquiries can be directed to the corresponding authors.

## Supplementary Materials

The following supporting information can be downloaded at: https://www.mdpi.com/article/10.3390/biomedicines13123004/s1, Table S1: Full datasets of differentially expressed proteins.

## Abbreviations

The following abbreviations are used in this manuscript:

AMD	Age-related macular degeneration
FC	Fold change
FDR	False discovery rate
LC-MS/MS	Liquid chromatography–mass spectrometry
LDL	Low-density lipoprotein
OxLDL	Oxidized low-density lipoprotein
PCA	Principal component analysis
RPE	Retinal pigment epithelium
TEAB	Triethylammonium bicarbonate
UPS	Ubiquitin–proteasome system
